# Development of a Green, Quick, and Efficient Method Based on Ultrasound-Assisted Extraction Followed by HPLC-DAD for the Analysis of Bioactive Glycoalkaloids in Potato Peel Waste

**DOI:** 10.3390/foods13050651

**Published:** 2024-02-21

**Authors:** Isabel Martínez-García, Carlos Gaona-Scheytt, Sonia Morante-Zarcero, Isabel Sierra

**Affiliations:** 1 Departamento de Tecnología Química y Ambiental, E.S.C.E.T, Universidad Rey Juan Carlos, C/Tulipán s/n, 28933 Móstoles, Spain; isabel.martinezg@urjc.es (I.M.-G.); cf.gaona.2019@alumnos.urjc.es (C.G.-S.); 2 Instituto de Tecnologías para la Sostenibilidad, Universidad Rey Juan Carlos, C/ Tulipán s/n, 28933 Móstoles, Spain

**Keywords:** GAs, potato peels, biovalorization, ultrasound-assisted extraction, HPLC-DAD

## Abstract

α-Solanine and α-chaconine are the two most predominant glycoalkaloids (GAs) present in potato. Potato peel contains a high concentration of GAs, which are especially interesting for application in the pharmaceutical industry due to their different beneficial properties (such as anticarcinogenic, anti-inflammatory, antiallergic, antipyretic, antiviral, fungicide, and antibiotic activities, among others); so, potato peel waste can be valorized by extracting these biologically active compounds. For this, a green, quick, and efficient miniaturized analytical approach based on ultrasound-assisted extraction (UAE) combined with HPLC-DAD was developed to quantify α-solanine and α-chaconine in potato peel. Some parameters of the extraction were optimized, including the extraction method, the type of solvent, and the sample/solvent ratio, by a three-factor, three-level (3^3^) full factorial experimental design. The optimal extraction conditions were obtained with UAE using methanol as a solvent and a sample/solvent ratio of 1:10 (*w*/*v*, g/mL). The analytical greenness metric for sample preparation (AGREEprep) tool was used to assess the greenness of the methods used. The tool revealed an acceptable green analysis, with 0.61 points. The method was validated and applied to the evaluation of GAs in the peel of 15 commercial varieties of potato. The amount of glycoalkaloids found in the samples evaluated ranged from 143 to 1273 mg/kg and from 117 to 1742 mg/kg dry weight for α-solanine and α-chaconine, respectively. These results reveal the important variability that exists between potato varieties; so, their analysis is of great importance to select the most suitable ones for biovalorization (e.g., the Amandine and Rudolph varieties, with around 3000 mg/kg, in total, of both GAs). To provide higher stability to the peel during storage, freeze-drying or a medium-temperature drying process resulted preferable to avoid GA degradation. Overall, this study will contribute to the expansion of the future biovalorization of potato peel waste as well as provide a powerful analytical tool for GA analysis.

## 1. Introduction

**Figure 1:**
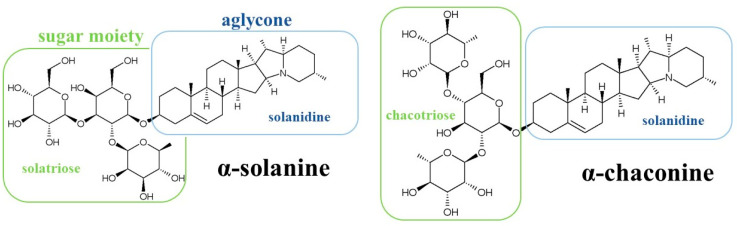
Chemical structures of α-solanine and α-chaconine.

Glycoalkaloids (GAs) are secondary metabolites found in plants, mainly of the Solanaceae family. They are nitrogen-containing glycosides that have a trisaccharide moiety attached, via the 3-OH group, to a lipophilic six-ring steroid aglycone skeleton (called solanidine) [1]. In the common cultivated potato (*Solanum tuberosum* L.), α-chaconine and α-solanine are the main GAs (Figure 1); together, they account for approximately 95% of the GAs found in potato; so, their total amount is generally referred to as total GAs (TGAs) [2].

GAs are present throughout the potato plant, including leaves, roots, flowers, fruits, tubers, and sprouts, with the highest levels observed in those parts of the plant with high metabolic rates [3]. In tubers, the distribution of GAs is not uniform, and their concentration is from 3 to 10 times greater in the peel than in the flesh, especially, in the green parts of the peel, eyes, and sprouts. In addition, large differences can be found in TGA concentrations in tubers depending on the potato variety and stage of maturity; however their accumulation is also affected by environmental variables during farming operation, postharvest handling, and storage, such as light exposure, high temperature, and mechanical damage, among others [4,5]. The presence of GAs in Solanaceae plants is associated with a natural defense mechanism against the attack of fungi, bacteria, insects, and herbivores. The ingestion of these compounds by humans at doses greater than 2 mg/kg of body weight can produce acute toxic effects due to the anticholinesterase action of GAs on the central nervous system, as well as cell membrane disruption, including gastrointestinal symptoms such as nausea, vomiting, diarrhea, and fever, and in more extreme cases, can cause neurological disorders, low blood pressure, coma, or death [6]. As a result, the EU in its Commission Recommendation 2022/561 on monitoring the presence of GAs in potatoes and potato-derived products established an indicative level of 100 mg/kg fresh weight (FW) of TGAs in potatoes and processed potato products [7]. However, although GAs are understood as potentially toxic, over the last two decades, some studies showed that they possess health-promoting effects. So, depending on the dosage and conditions of use, beneficial properties, such as anticarcinogenic, anti-inflammatory, antiallergic, antipyretic, antiviral, fungicide, and antibiotic activities, have been demonstrated, among others [8,9].

Nowadays, the potato stands as a significantly vital vegetable, cultivated and consumed across numerous countries worldwide, acting as high-quality food rich in carbohydrates, proteins, vitamins, minerals, and fiber [10,11]. Ranking as the third largest food crop, its global production reached 359.07 million tons in 2020, according to the Food and Agricultural Organization of the United Nations (FAO) [12]. Forecasts suggest its rise to a prominent position within the global food security framework, especially as other cereal crops approach their yield limits. It is noteworthy that two-thirds of the world’s population consider potatoes a staple food. Within developing countries, fresh potato remains the primary consumption preference, but the consumption of processed potato products such as potato French fries, potato chips, frosted potato, dehydrated potato flasks, etc., is gradually increasing [12]. This high consumption generates a huge volume of by-products, mainly potato peels, which are a problem, since wet peels are prone to microbial deterioration. This by-product is usually used as low-value animal feed or discarded, causing environmental concern. In fact, it is anticipated that in 2030 around 8000 kilotons of potato peel waste might be generated, with related greenhouse gas emissions of 5 million tons of CO_2_ equivalents [13]. In that respect, in an approach to the concept of “One Health”, potato peel waste can be valorized by extracting high-value-added compounds (i.e., phenolic compounds, GAs, fiber, minerals, etc.), particularly interesting for application in the food and pharmaceutical industries, that can be used for the formulation of nutraceuticals and phytopharmaceutical products or as additives and ingredients in functional foods [9,14]. For this reason, efficient and sustainable industrial-scale extraction processes to obtain high-value extracts are required, being this one of the greatest challenges to be addressed for potato peel waste valorization [9]. In addition, this involves the development and validation of analytical protocols that allow for the characterization of the composition of the potato peels before they can be released for potential biovalorization [8].

Nowadays, analytical methods should be aligned with the current trend towards green analytical chemistry (GAC) whose most important assumptions were formulated in the form of twelve principles that express a willingness to care for human safety and the environment during the development and application of analytical procedures [15]. In this regard, the proper implementation of the GAC principles would include practices such as minimizing reagent consumption, use of biodegradable and low-toxic reagents, saving energy, reducing waste production, and increasing the degree of miniaturization of analytical tools and procedures, among others. Regarding GA analysis, high-performance liquid chromatography with a diode array detector (HPLC-DAD) or coupled to mass spectrometry (HPLC-MS) are recommended techniques [6] that have traditionally been used after GA extraction with an appropriate sample treatment protocol. Despite MS detection having advantages (i.e., higher selectivity), HPLC-MS methods require an extract purification step to avoid matrix effects that is time-consuming. For this reason, HPLC-DAD should be considered a suitable alternative due to the relative widespread availability of the required instrumentation in analytical laboratories. Conventionally, α-solanine and α-chaconine are extracted from potato peel using the solid–liquid extraction (SLE) technique, using different laboratory mixers (i.e., vortex mixers, magnetic stirrers, shakers) to improve the extraction rate [16]. On the other hand, ultrasound-assisted extraction (UAE) has also been evaluated as, in general, this technique notably reduces extraction times and energy and solvent consumption and enhances the recovery yields of the target analytes [17]. In this sense, UAE is a potential environmentally friendly choice to extract GAs from potato peels, which can be scaled up to the industrial level [18]. However, for an efficient GA extraction in line with GAC, some parameters need to be optimized, and the analytical methodology has to be validated to assure the quality of the results. In addition, because the efficiency of this extraction process depends on several parameters, to achieve the optimal experimental conditions, a design of the experiments is advisable. The methods developed would be useful to find and use potato varieties that have a greater or lesser tendency to accumulate GAs in the peel, depending on the final use of the extract obtained. In addition, the discovery of health benefits of potato GAs, balanced against concerns of their toxicity, implies that the analytical methodology will be paramount in future efforts designed to enhance the levels of these compounds in the human diet [11].

Therefore, the aim of this study was to compare different extraction protocols for α-solanine and α-chaconine from potato peel waste. Some parameters of the extraction were optimized, including the extraction method, the type of solvents, and the sample/solvent ratio by a three-factor and three-level (3^3^) full factorial experimental design methodology. Under optimal conditions, the performance of the UAE-HPLC-DAD developed method was evaluated and applied to determine the levels of α-solanine and α-chaconine in the peel of fifteen varieties of potato. Finally, the effect of temperature on the GA content in the peel extracts was evaluated with the developed procedure. To the best of our knowledge, this is the first green, quick, efficient, and validated analytical methodology based on HPLC-DAD that allows the characterization of the main GAs in this by-product.

## 2. Materials and Methods

### 2.1. Reagents and Materials

The HPLC-grade solvents such as methanol (MeOH) and ethanol (EtOH) were obtained from Scharlab (Barcelona, Spain), and acetonitrile (ACN) was obtained from Fisher Chemical (Madrid, Spain). Monosodium phosphate (NaH_2_PO_4_) and disodium phosphate (Na_2_HPO_4_) were purchased from Panreac (Barcelona, Spain). Ultrapure deionized water with a resistance of 18.2 MΩ cm was obtained by a Milli-Q system (Billerca, MA, USA). Nylon syringe filters (0.45 μm, 13 mm) were purchased from Scharlab (Barcelona, Spain). For standard solutions preparation, α-solanine (>99%) and α-chaconine (>99%), purchased from Phytolab (Madrid, Spain), were used. Individual stock standard solutions of α-solanine and α-chaconine were prepared at a concentration of 200 μg/mL in MeOH and stored at −20 °C. The calibration standards were prepared at the appropriate concentrations (1, 2.5, 5, 10, 25, 50, and 100 mg/L for both analytes) by serial dilution of the stock solutions in MeOH. The prepared solutions were stored in the dark at a temperature of −20 °C.

### 2.2. Sample Collection and Moisture Determination

Fifteen commercial varieties of potato (Agata, Agria, Amandine, Amaris, Caesar, Colomba, Evolution, Frisia, Lady Amarilla, Memphis, Monalisa, Rudolph, Soprano, Universa, and Vivaldi) were acquired from supermarkets in Madrid and Albacete (Spain) in 2023. Table S1 summarizes the labelling information found for the prepackaged fresh potatoes used in this study (e.g., origin, caliber, category, and recommended use). The potatoes were peeled manually with a knife peeler (~1.5 mm thickness). The peels were then pre-frozen in an ultra-freezer at −80 °C for 24 h and then freeze-dried in a LyoBench freeze-dryer (Noxair Life Sciences S.L., Barcelona, Spain) for 48 h at a temperature of −50 °C and a pressure of 0.076 mbar. Subsequently, the freeze-dried samples were powdered using an analytical grinder (IKA, Staufen, Germany) to yield potato peel powder, which was stored in Falcon^®^ tubes into a desiccator at room temperature until use. The total moisture content in the peels was found to be between 78 and 85% (Table S1), determined by loss on oven-drying at 60 °C for 24 h, as described [19]. In the optimization of GA extraction, all experiments were carried out with the Caesar potato variety. Due to the content of GAs not being homogeneous in potato peel, for all the optimization experiments, a quantity of homogenized and powdered sample large enough to be used in all of them was prepared.

### 2.3. Optimization of the Sample Treatment Conditions and Statistical Analysis to Maximize the Extraction Yield of α-Solanine and α-Chaconine

#### 2.3.1. Preliminary Studies

Preliminary studies were conducted to assess the efficacy of different solvents and extraction methods in the extraction of GAs. First, the sample and solvent (in a ratio of 1:20, *w*/*v*, g/mL) were homogenized by vortexing for 1 min at 3000 rpm (Rx3 Velp Scientifica, Usmate, MB, Italy). Then, for conventional SLE, the mixtures were subjected to agitation for 5, 10, 15, and 20 min with a magnetic stirrer (IKA RCT basic, Staufen, Germany). On the other hand, for UAE, the mixtures were sonicated with a Sonopuls HD 3100 ultrasonic homogenizer (100 W, 60 kHz, Bandelin, Berlin, Germany) equipped with an MS 73 titanium probe (13 mm diameter). The UAE employed an amplitude of 75% in pulsed mode (pulse durations of 0.1 s “on” and 0.2 s “off”) for 5, 10, 15, and 20 min. These experiments were carried out at ambient temperature (around 23 °C) and were performed in triplicate.

#### 2.3.2. Design of Experiments to Reach the Optimal Extraction Conditions

A full factorial experimental design methodology employing three factors at three levels (3^3^) was utilized. This design aimed to assess the impact of the extraction method (A), solvent type (B), and sample/solvent ratio (C) on GA extraction. The extraction methods examined in the experimental design included (i) conventional vortex-assisted SLE (VA-SLE) for 1 min; (ii) conventional SLE with magnetic stirring (MgS-SLE) for 5 min; (iii) UAE with 5 min of sonication. The solvents tested were MeOH, EtOH, and H2O, while the sample/solvent ratios of 1:10, 1:20, and 1:40 (*w*/*v*, g/mL) were evaluated. The choice of the levels for each independent variable was based on preliminary experiments and previous related research. The experimental design, the analysis of the results, and the prediction of the responses were conducted using Statgraphics Centurion software (version 16.3.03).

#### 2.3.3. Optimized Extraction Conditions

For UAE of GAs, 0.3 g of a sample (freeze-dried potato peel powder) was placed in a Falcon^®^ tube, and 3 mL of MeOH was added (sample/solvent ratio of 1:10 *w*/*v*, g/mL). The ultrasound probe was submerged to a depth of 5 mm in the solvent, and the mixture was sonicated at a constant frequency for 2.5 min at room temperature. Then, 1 mL of the resulting solution was filtered through a nylon membrane (0.45 μm) and, subsequently, analyzed by HPLC-DAD. The extraction was performed in triplicate (n = 3), and the results are presented as mean ± standard deviation (SD).

### 2.4. Optimal Chromatographic Conditions for Analysis

The chromatographic analysis was performed on an Agilent 1260 Infinity II HPLC system (Agilent Technologies, Madrid, Spain), equipped with a G7104C 1260 flexible pump, a G7167A multisampler, a G7116A multicolumn thermostat, and a G7117C diode array detector HS. Agilent OpenLab CDS ChemStation Edition was used for full instrument control and data acquisition and analysis. The separation of α-solanine and α-chaconine was performed using an InfinityLab Poroshell 120 EC-C18 column (3.0 mm i.d. × 150 mm, 2.7 μm particle size) to which a guard column (3.0 mm i.d. × 50 mm, 2.7 μm particle size) with the same stationary phase was attached. The mobile phase was a mixture of ACN–0.01 M sodium phosphate buffer, pH 7.2–MeOH (60:30:10, *v*/*v*/*v*) in isocratic mode. The flow rate was set at 1 mL/min with an injection volume of 20 μL. The column temperature was maintained at room temperature, and the autosampler tray was cooled to 4 °C. The analysis time was 11 min. Quantification was performed by UV detection at 202 nm and comparison with the external standards for both compounds. The results are expressed as mg/kg dry weight (DW) of each analyte and as TGAs (sum of α-solanine and α-chaconine levels).

### 2.5. Method Validation

Due to the current lack of no official regulations on analytical performance requirements for GAs in food, method validation was conducted in terms of linearity, method detection (MDL) and quantification (MQL) limits, accuracy, precision, and selectivity, following the criteria described in the SANTE/12682/2019 document in regulation EC No 401/2006 and in the Q2(R1) ICH guidelines [20,21,22]. Linearity was evaluated through calibration curves, which were constructed using five standard solution mixtures containing from 1 to 100 mg/L of each GA for three consecutive days. A suitable regression analysis of the signal (y, peak area) on the analyte concentrations (x) established in the calibration set yielded the calibration curve for the predicted responses. Linearity was evaluated through the coefficient of determination (R^2^) of the calibration curves. The sensitivity of the method was determined through the LOD and LOQ from the analysis of the least concentrated standard solution analyzed (1 mg/L), which were estimated as the lowest concentrations of analyte that could be detected and quantified with a signal-to-noise ratio (S/N) exceeding 3 and 10, respectively. The accuracy was evaluated by spiking the samples at low (200 mg/kg of each analyte) and high (400 mg/kg of each analyte) levels, obtaining the recovery values (% ± SD). Recoveries were calculated by comparing the areas obtained for samples spiked with a known concentration of the target analytes and subjected to the UAE optimized procedure (n = 6) with those areas obtained for the simulated samples (samples that were spiked at the same concentration but at the end of the UAE procedure, just prior to their chromatographic analysis). The recovery values had to be between 70 and 120%. On the other hand, the method precision was evaluated in terms of repeatability and reproducibility, using the same validation levels (200 and 400 mg/kg of each analyte). Repeatability is expressed as the relative standard deviation (RSD, %) for six replicates (n = 6) of a sample spiked with the GAs, at the low and high validation levels, in the same day. Reproducibility (also expressed as %RSD) was calculated by the analysis of three replicates of a sample (spiked with the analytes at both validation levels), which was carried out over three different days (n = 9). According to the validation guidelines used, the RSD values for these precision parameters had to be ≤20%. The selectivity of the method was determined by comparing the UV–Vis spectra of standard solutions of α-solanine and α-chaconine with the spectra obtained for the target analytes in the spiked and non-spiked sample extracts. The absence of coeluted peaks and signals from matrix interferences, as well as the constant retention times, revealed the selectivity of the method.

### 2.6. Evaluation of the Effect of the Drying Conditions on the α-Solanine and α-Chaconine Content in Potato Peels

To assess the effect of the temperature during potato peel drying on the GA content, two heating conditions achieved using a conventional laboratory oven (60 °C and 103 °C for 24 h) were evaluated. The potato peels from six different varieties were subject to drying (Agata, Amandine, Caesar, Monalisa, Rudolph, and Soprano). Subsequently, once the TGA content in each potato peel had been analyzed, the results were compared to those obtained for the same samples subjected to freeze-drying, as indicated in Section 2.2.

## 3. Results and Discussion

### 3.1. Optimization of the Chromatographic Method for α-Solanine and α-Chaconine Determination

**Figure 2:**
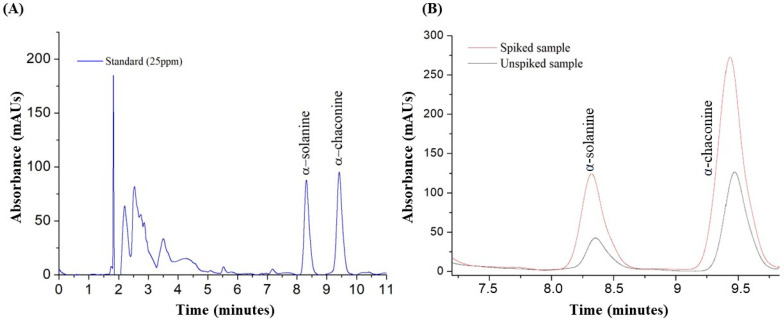
(**A**) Chromatographic separation of a standard solution of α-solanine and α-chaconine in MeOH. (**B**) Chromatograms of non-spiked and spiked Agata variety samples (40 mg/L). Both chromatograms are shown at 202 nm using a mobile phase comprising 60% ACN, 30% phosphate buffer, and 10% MeOH.

The separation of α-solanine and α-chaconine can be achieved by HPLC-DAD on reversed-phase mode with C18 columns using aqueous phosphate buffers in combination with an organic modifier, typically MeOH or ACN [6]. Taking this into account, various experiments were conducted to optimize the chromatographic parameters. This included assessing the ratio between organic solvents (ACN or MeOH) and the aqueous phosphate buffer as the mobile phase. Based on previous work [23], a combination of 60% ACN and 10% MeOH as the organic solvent was selected. In regard to the aqueous solution, a 30% solution of sodium phosphate buffer was evaluated at different pH values. The results obtained revealed that the best results were achieved with a 0.01 M phosphate buffer solution at pH 7.2. Further optimization included adjusting the column temperature to 20 °C and setting the flow rate to 1 mL/min. In this optimized conditions, α-solanine exhibited a retention time of 8.3 min, while α-chaconine showed a retention time of 9.4 min, achieving a resolution of 5.1. This indicated an exceptional chromatographic separation of the target analytes (Figure 2).

### 3.2. Optimization of the Extraction Methodology of α-Solanine and α-Chaconine from Potato Peels

#### 3.2.1. Preliminary Studies

**Figure 3:**
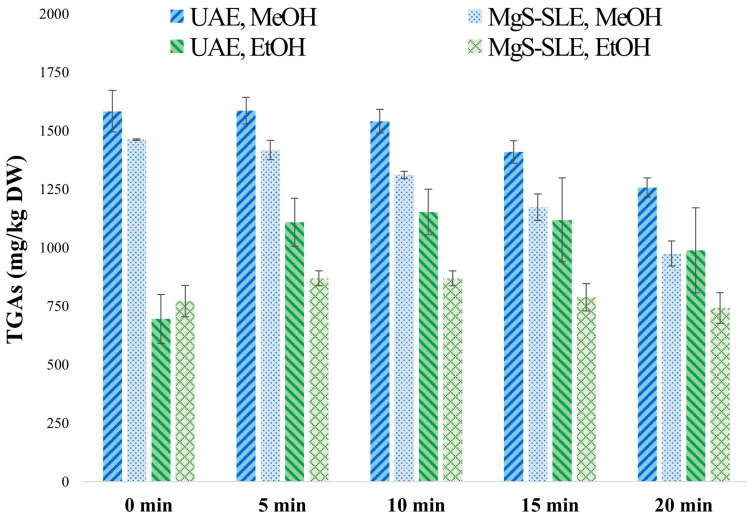
TGAs determined in potato peel after solid–liquid extraction with magnetic stirring (MgS-SLE) and ultrasound-assisted extraction (UAE) for different times, using ethanol (EtOH) or methanol (MeOH) as the extraction solvent. The sample–solvent mixture (1:20, *w*/*v*) was first subjected to vortex homogenization and then analyzed (0 min).

Preliminary experiments were carried out using the one-factor-at-a-time methodology to select the variables for the experimental design to optimize the extraction process. Figure 3 shows the results obtained expressed in mg of TGAs per kg of DW. For this purpose, 0.5 g of sample was weighed, and 10 mL of solvent (sample/solvent ratio 1:20, *w*/*v*, g/mL) was added. The extractions were performed with both MeOH and EtOH, since they are common solvents previously used by other authors [24,25,26,27,28]. In all experiments, the sample–solvent mixture was first subjected to 1 min of vortex agitation in order to disperse the sample into the solvent and to accelerate the extraction process; then, the resulting extracts were analyzed (0 min in Figure 3). The extracts were subjected to MgS-SLE or UAE. In both cases, 1 mL aliquots were taken every 5 min, and the TGA concentration was determined in the extracts (5, 10, 15, and 20 min, in Figure 3). Based on the results obtained in previous work by Apel et al. [16], all experiments were carried out with UAE in pulse mode with an amplitude of 75%, since these conditions did not have any significant effect on the extraction yields of individual GAs and on the TGAs of potato peels, with pulse amplitude in the range of 50–100%. The continuous-pulse mode was not tested, as it involves higher energy consumption. Furthermore, experiments were conducted at room temperature with the aim of developing a potential low-cost, green protocol that could be suitable for industrial-scale extraction.

As shown in Figure 3, the highest value of TGAs (around 1600 mg/kg DW) was achieved with MeOH. Using this solvent, no significant differences were found in the extraction after applying 5 min of magnetic stirring or UAE, compared to vortexing exclusively (0 time, Figure 3). On the other hand, with EtOH, it was observed that 5 min of magnetic stirring or UAE produced an increase in the TGAs extracted, with a higher value obtained with UAE. Extraction times greater than 5 min did not increase the extraction yields of the target analytes with either of the two solvents. Whit these results in mind, VA-SLE (for 1 min), MgS-SLE (for 5 min), and UAE (for 5 min) were selected to be evaluated in the experimental design to optimize the extraction protocol.

Taking into account the results obtained with EtOH, which is a greener and more environmentally sustainable extraction solvent, assays were additionally carried out to evaluate whether the increase in temperature improved the extraction of GAs, as this is a more viscous solvent. The tests were carried out at 50 °C applying 1 min of VA-SLE or 5 min of UAE, and the results showed no significant increase in the extraction yields. For this reason, this variable was not included in the experimental design aimed at developing a potentially low-cost and environmentally friendly protocol that could be suitable for industrial-scale extraction.

On the other hand, for the selection of a third solvent, SLE and UAE experiments were carried out with H_2_O, CH_3_COOH (1 and 5%), 5% CH_3_COOH–MeOH (1:1 and 1:4, *v*/*v*), ACN, 0.01 M sodium phosphate buffer (pH 7.2), and ACN–0.01 M sodium phosphate buffer (pH 7.2)–MeOH (60:30:10, *v*/*v*/*v*). Among these options, considering that the extraction yields were not significantly improved with any of the solvents tested, H_2_O was selected for the experimental design, as it is a more environmentally sustainable option. Furthermore, the aqueous extracts were suitable for HPLC analysis, allowing for achieving a very good chromatographic resolution of the α-solanine and α-chaconine peaks.

Finally, in order to maximize the extraction of TGAs and to minimize the amount of residues, the sample/solvent ratio was considered as the third independent variable for the experimental design. In that respect, some preliminary assays were carried out to verify the influence of this variable on TGA extraction (from 1:10 to 1:40, *w*/*v*, g/mL), with significant differences between the different types of extraction and solvents used.

#### 3.2.2. Experimental Design, Evaluation of the Variables Influencing the Extraction Efficiency, and Statistical Analysis

The optimization of the extraction conditions by the one-factor-at-a-time methodology does not consider the possible interactions between the studied factors. Therefore, it is very useful to use the full factorial experimental design methodology that allows the number of experiments to be minimized and the effect of each factor and the interaction between the factors on the extraction yield to be evaluated simultaneously. Based on the results obtained from the preliminary tests, the full factorial experimental design proposed in this work included three independent variables, corresponding to the three different factors of the design, two of them being categorical (extraction type (A) and solvent type (B)), and the other numerical (sample/solvent ratio (C)). Each of them had three different levels: the categorical ones had level 1, 2, and 3, while the numerical one had a low (−1), a medium (0), and a high (1) level. The dependent variables corresponded to the concentrations of the analytes, i.e., TGAs, α-solanine, and α-chaconine, obtained in each case (Table 1).

Table 2 shows the results obtained for the 27 assays indicated in the experimental design matrix (Table 1), expressed as mean concentration values obtained for three replicates ± SD. As can be seen, the experimental values obtained ranged from 15 ± 6 to 917 ± 75 mg/kg DW for α-solanine, from 24 ± 7 to 915 ± 42 mg/kg DW for α-chaconine, and from 48 ± 22 to 1622 ± 157 mg/kg DW for TGAs.

**Figure 4:**
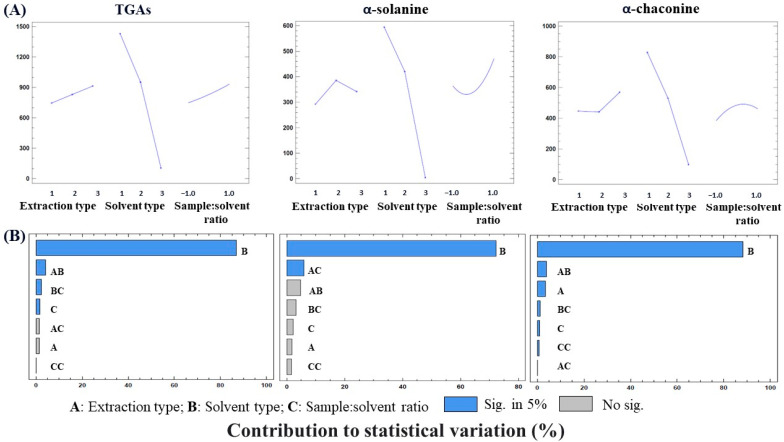
(**A**) Main effects chart of the three examined variables (extraction type, solvent type, and sample/solvent ratio) at three levels (see Table 1) for three responses (contents of TGAs, α-solanine. and α-chaconine, mg/kg DW). (**B**) Pareto charts from the 3^3^ full factorial experimental design of the standardized effect on each of the responses (contents of TGAs, α-solanine, and α-chaconine, mg/kg DW) for the analysis of the three variables (A) extraction type; (B) solvent type; (C) sample/solvent ratio. *p* value is significative when it is <0.05.

To determine the effects of each variable and the possible interactions between them, both the main effects plot of each variable and the Pareto chart were constructed and are showed in Figure 4. The main effects plot can be used to compare the relative strength of the effect of different variables as well as to determine the positive or negative effect of each variable on the response. The Pareto chart shows the absolute values of the standardized effects of each variable and the possible interactions between them and allows for determining whether a factor has a significative effect on the response. The gradients of the main effects plot shown in Figure 4A indicated that the type of solvent (B) was the most influential parameter with the same effect on α-solanine and α-chaconine. The extraction type (A) and the sample/solvent ratio (C) showed a lower effect on the GA extraction yield, which was opposite for α-solanine and α-chaconine. While for α-solanine the best type of extraction was MgS-SLE, for α-chaconine, the best type was UAE, making this latter type of extraction the best option for the analysis of TGAs. In relation to the sample/solvent ratio (C), the highest extraction yield for α-chaconine was obtained with the sample/solvent ratio of 1:20, while for α-solanine, the extraction increased by increasing the sample/solvent ratio up to 1:40, resulting in a slightly positive effect of this variable on the extraction of TGAs.

The trend observed in the main effects plot (Figure 4A) for the three variables coincided with that observed in the Pareto chart (Figure 4B). As can be seen, the solvent type (B) was the variable which had the strongest influence, showing a much more significant effect on the GA extraction yield than the rest of the variables (A and C). This fact could be due to the intrinsic chemical nature of the solvents used. Water, being a high-polarity solvent, had a lower GA extraction efficiency, since aglycones have a lipophilic nature. In contrast, MeOH and EtOH, being less polar organic solvents than water, allowed for a better extraction efficiency of α-solanine and α-chaconine. The effect observed for the extraction type (A) was only significant for the α-chaconine extraction yield, and the effect of the sample/solvent ratio (C) was significant for the α-chaconine and TGAs extraction yields.

Regarding the interaction between the three variables studied, the interaction between extraction type and solvent type (AB) was considered significant for the α-chaconine and TGA extraction yields; so, it can be deduced that each solvent was efficient to a greater or a lesser extent depending on the type of extraction used. α-Chaconine and TGAs were also significantly affected by the interaction between solvent type and sample/solvent ratio (BC), whereas the interaction between extraction type and sample/solvent ratio (AC) had a significant effect only on the α-chaconine extraction yield. Finally, the quadratic value of the sample/solvent ratio (CC) was not significantly influential in any of the cases, showing that this variable did not exert a significant effect on the GA extraction yield.

The statistical parameters obtained from the statistical analysis (ANOVA) (F-values, *p*-values, R^2^, R^2^ adjusted, R^2^ predictive) are reported in Table S2 and indicated that the resulting quadratic models had very high predictability and could be used to optimize the extraction procedure of GAs from potato peel. The statistical analysis also confirmed the significant terms of the obtained quadratic models (*p* < 0.05) showed in the Pareto chart (Figure 4B).

Based on the statistical outcomes derived from the full factorial experimental design, an optimized extraction procedure for obtaining the highest extraction efficiency of GAs from potato peel samples was established. Statistically, it was deduced that the most efficient extraction type (A) was UAE (5 min) with MeOH as the extraction solvent (B) and a sample/solvent ratio (C) of 1:10 (*w*/*v*, g/mL). To verify the reliability of the statistically estimated optimum, the experimentally obtained results were compared with the predicted quantitative results. To maintain the sample/solvent ratio while reducing waste production and reagent consumption, extractions were performed with 0.3 g of sample and 3 mL of MeOH, as the ultrasonic probe used in the laboratory had a recommended sample volume range of up to 3 mL. Table 3 demonstrates a high similarity between the experimental and the predicted results, validating the effectiveness and reliability of the optimized method for determining the optimal extraction conditions of GAs and maximizing their extraction.

**Figure 5:**
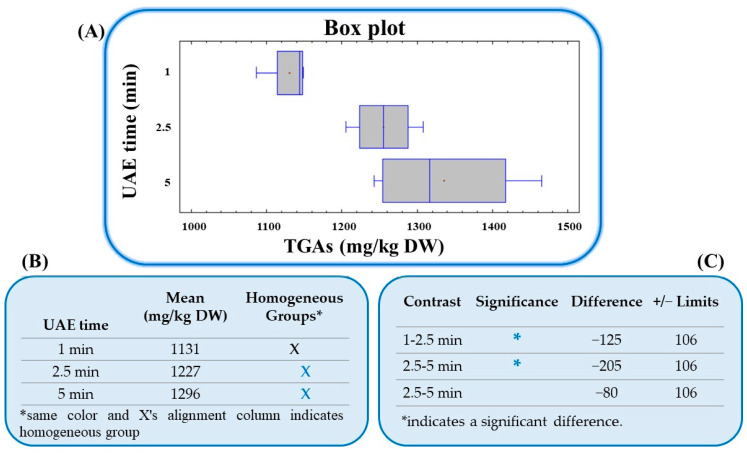
(**A**) Box plot. (**B**) Homogeneous group values obtained from the Fisher’s multiple range test. (**C**) Significant differences between pair values obtained from the Fisher’s multiple range test demonstrating the statistical differences for the three extraction times evaluated under UAE optimized conditions for TGA determination (combined amounts of α-solanine and α-chaconine) in potato peel. All results were obtained with 95% confidence.

Finally, once the optimal extraction conditions obtained for GAs to maximize their extraction were established, a re-optimization study was carried out to determine if the extraction time could be reduced and increase the sustainability of the developed method. For this purpose, new UAE experiments were carried out applying 1, 2.5, and 5 min of sonication. All assays were performed on the same day in triplicate (n = 3) under the optimal conditions. Fisher’s least significant difference (LSD) test was performed to discriminate between the mean TGA concentrations determined, which were 1131 ± 30, 1227 ± 50, and 1296 ± 69 mg/kg DW after 1, 2.5, and 5 min, respectively. As it can be seen in Figure 5, the boxplot shows visually the distribution of the obtained data and demonstrated that there were significant differences between the GA amounts extracted in the first (1 min) and second trials (2.5 min), but there were no significant differences between those extracted in the second (2.5 min) and third trials (5 min). Additionally, the data shown in Figure 5B indicate two homogeneous groups according to the alignment of X in the columns: one group for trial 1 (1 min), and the other homogeneous group for trials 2 (2.5 min) and 3 (5 min). This means that there were no statistically significant differences between the values obtained after 2.5 and 5 min of sonication. In Figure 5C, we applied a multiple comparison procedure to determine which means were significantly different from others. The asterisks showed in the pairs 1–2.5 min and 1–5 min indicate that these pairs showed statistically significant differences at the 95% confidence level, whereas the pair 2.5–5 min did not show statistically significant differences at the same confidence level. Therefore, given that with 2.5 min of sonication the capacity to extract the maximum amount of GAs from potato peel samples was reached, this was the optimal sonication time chosen for the method validation.

### 3.3. Method Validation

The optimized UAE-HPLC-DAD method for the quantification of GAs in potato peels was validated, and the results are shown in Table 4. The external calibration curves (1–100 mg/L) were obtained with an R^2^ ~ 0.991 for both analytes. In addition, the deviation of the slopes of the calibration curves obtained on three different days and with three consecutive injections for each standard solution (n = 9) was calculated obtaining RSD between 2 and 18%. The values obtained for LOD and LOQ were 0.3 mg/L and 1 mg/L, for both analytes analyzed, respectively. Accuracy was evaluated at two different concentration levels, showing adequate mean recovery values of 103 ± 5 and 100 ± 4% for α-solanine and α-chaconine, respectively (Table 4). On the other hand, as shown in Table 4, satisfactory results were obtained for intra-day and inter-day precision at the two concentration levels, since the RSD values were lower than 13%. Finally, the selectivity of the method was also studied, as shown in Figure 2, where the chromatograms obtained for the standard solutions of α-solanine and α-chaconine are compared with spiked and non-spiked sample extracts. Furthermore, a determination of selectivity was made by assessing the purity of the chromatographic peaks. To achieve this, the absorption spectra of α-solanine and α-chaconine were obtained, with no evidence of co-elution of any other compounds or interferents at the retention time of the target analytes. In addition, the retention times showed a deviation ≤ 0.1 min for all the analytes.

### 3.4. Greenness Evaluation of the Developed Method

**Figure 6:**
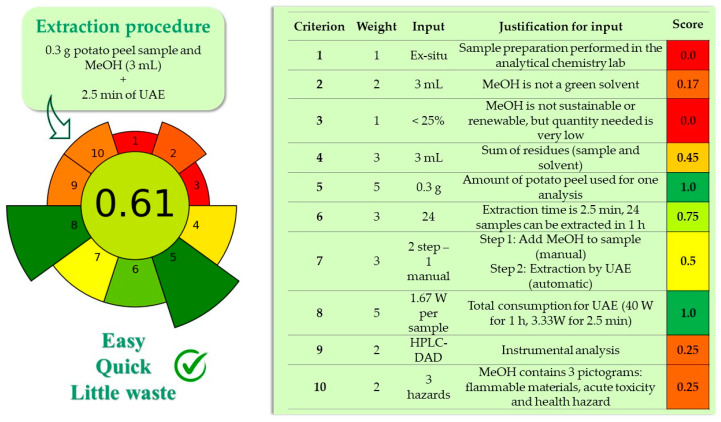
Greenness results of the UAE-HPLC-DAD method developed for glycoalkaloid determination in potato peel evaluated with the AGREEprep metric. MeOH: methanol; UAE: ultrasound-assisted extraction.

The analytical procedure developed for the extraction of GAs from potato peel was evaluated in terms of greenness using the analytical greenness metric for sample preparation (AGREEprep) [29]. Using this tool, the overall sample preparation greenness performance is indicated by a pictogram with an inner circle representing the overall sample preparation score and a color based on traffic lights (from red to green). The criteria used were based on the ten principles of green sample preparation (e.g., use safe solvents and reagents, minimize waste, maximize sample throughput, etc.) [30], whose overall values can range from 0 to 1, the score of 1 indicating greenness performance (see Figure 6). As it can be observed, the proposed analysis method achieved an optimal score of 0.61 points due to the sample and solvent miniaturization and a significant reduction in the extraction time, calculated according to the criteria and scores established for this method (Figure 6).

### 3.5. Application to the Analysis of Different Potato Peels

The methodology developed was applied to determine the concentration of α-solanine and α-chaconine in the peel waste of fifteen different commercial varieties of potatoes (Table S1). It can be seen in Table 5 that the TGA concentration in potato peel waste ranged from 260 ± 8 to 2823 ± 33 mg/kg DW, being Vivaldi the potato variety with the lowest content, and Amandine the one with the highest content. In addition, significant differences in the TGA content in the potato peels were found depending on the variety analyzed. Nevertheless, it remains uncertain whether the concentration of GAs in the samples analyzed was predominantly influenced by the potato variety or by other variables related to their cultivation and storage conditions. This is because the samples were chosen at random on the market; so, the external factors that affect the GA content (such as climate, soil type, light exposure, maturity, etc.) were not controlled in this study. These results agree with those found in the literature, and according to [2], depending on the potato cultivar, irradiation, storage conditions, and mechanical injury, the TGA content in potato peel can ranged from 84 to 3526 mg/kg DW.

As it can be seen in Table 5, α-chaconine was generally found in a higher percentage (from 45 to 77%) than α-solanine (from 23 to 55%), except in the Vivaldi potato variety, where 55% of α-solanine and 45% of α-chaconine were found. These results are consistent with those of other authors such as Musita et al. [31], who typically found a higher quantity of α-solanine than of α-chaconine. The concentration of α-solanine in the potato peel varied between 143 and 1273 mg/kg DW, while the concentration of α-chaconine varied between 117 and 1742 mg/kg DW. There seemed to be no differences between potatoes with a yellow or with a red peel and neither between potatoes of large nor small caliber (Table 5). In this sense, these results demonstrated that the proposed UEA-HPLC-DAD method is suitable to determine GAs in potato peel samples at concentrations varying in a wide range.

### 3.6. Evaluation of the Temperature Effect on the Concentration of α-Solanine and α-Chaconine during Drying

**Figure 7:**
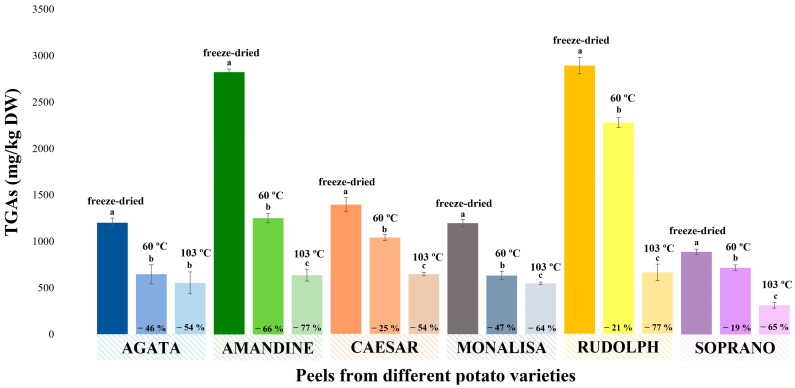
Effect of the heating temperature during drying compared to freeze-drying in different potato peel samples. TGAs refers to the combined amounts of α-solanine and α-chaconine. Different letters (a, b, c) means statistically significant differences (*p* ≤ 0.05).

Figure 7 shows the effect of the temperature during potato peel drying on the GA content. As indicated in Section 2.6, two heating conditions, achieved using a conventional laboratory oven (60 °C and 103 °C for 24 h), were evaluated, and the results were compared to those obtained for the same samples subjected to freeze-drying. It was observed that the drying temperature had a significant effect on the TGA content in the potato peel samples. Thus, in all samples analyzed, the content of TGAs decreased significantly when the peels were subjected to heat drying. The decrease in TGAs at 60 °C varied from 20% (Soprano) to 62% (Amandine), while degradation at 103 °C resulted in a reduction in TGAs from 54% (Agata, Caesar and Monalisa) to 77% (Rudolph and Amandine), as shown in Figure 7. As observed, in general, the level of analyte reduction increased with the heating temperature. Since potato peels have a high moisture content, they require a drying treatment to extend their shelf life, avoiding enzymatic and microbial degradation during storage. In this sense, the results obtained in this study are important, since it was observed how the drying conditions affect the GA content. Therefore, to obtain extracts at an industrial level rich in these compounds (for subsequent purification and GA isolation) it would be preferable to use freeze-drying or a medium-temperature drying process.

## 4. Conclusions

A green, quick, and efficient miniaturized analytical approach using HPLC-DAD was developed to quantify α-solanine and α-chaconine in potato peel discarded by the food industry and consumers. A statistical analysis was carried out through a design of experiments (3^3^) to maximize the extraction of the target compounds in potato peels. The parameters optimized were the extraction method (conventional vortex-assisted SLE, conventional SLE with magnetic stirring, and UAE), the type of solvent (MeOH, EtOH, and H_2_O), and the sample/solvent ratio (1:10, 1:20, and 1:40, *w*/*v*, g/mL). The optimal extraction conditions involved the use of MeOH as a solvent, the application of the UAE technique for 2.5 min, and a sample/solvent ratio of 1:10, *w*/*v*. The AGREEprep tool used to evaluate the method indicated its acceptable ecological character, with a score of 0.61 points. The developed method was successfully validated and demonstrated an excellent recovery of the target analytes (around 100%), low limits of quantification (1 mg/L, for both analytes), good precision, and selectivity. In addition, it was applied to 15 varieties of commercial potato peels to determine their GA concentration, yielding values of up to 2895 mg/kg DW, for the combined amounts of both compounds in the Amandine and Rudolph varieties. Given the high levels of TGAs found in commercial samples, potato peels could potentially be valorized through the extraction of high-value-added compounds and used in the development of new products for the pharmaceutical industry. Lastly, it was verified that controlling the drying temperature is crucial, as the compounds to be extracted are affected. If scaled up to food industry levels, it is advisable to use controlled temperatures as low as possible to prevent the degradation of the GAs of interest.

## Figures and Tables

**Table 1:** Summary of the three independent factors and their three different levels used for the experimental design.

Factor Type	Symbol	Factor Levels		
Categorical		Type 1	Type 2	Type 3
Extraction type	A	VA-SLE 1 min	MgS-SLE 5 min	UAE 5 min
Solvent type	B	MeOH	EtOH	H_2_O
Numerical		Low (−1)	Medium (0)	High (+1)
Sample/solvent ratio (*w*/*v* (g/mL))	C	1:10	1:20	1:40

EtOH: ethanol; MeOH: methanol; MgS-SLE: solid–liquid extraction with magnetic stirring; VA-SLE: vortex-assisted solid–liquid extraction.

**Table 2:** Results obtained from the 3^3^ full factorial experimental design methodology to optimize the glycoalkaloid extraction conditions from potato peel.

RUN	Factor *			Responses (Mean Concentration, mg/kg DW ± SD)		
	A	B	C	α-Solanine (Y_1_)	α-Chaconine (Y_2_)	TGAs (Y_3_)
1	1	1	−1	748 ± 13	818 ± 38	1566 ± 41
2	1	1	0	658 ± 44	876 ± 54	1584 ± 88
3	1	1	1	482 ± 12	726 ± 58	1208 ± 47
4	1	2	−1	238 ± 28	179 ± 13	417 ± 41
5	1	2	0	283 ± 46	424 ± 54	696 ± 104
6	1	2	1	421 ± 89	471 ± 50	892 ± 85
7	1	3	−1	58 ± 22	63 ± 18	121 ± 27
8	1	3	0	111 ± 18	51 ± 5	162 ± 20
9	1	3	1	94 ± 7	39 ± 3	132 ± 9
10	2	1	−1	528 ± 21	546 ± 25	1074 ± 43
11	2	1	0	456 ± 30	768 ± 33	1225 ± 37
12	2	1	1	917 ± 78	646 ± 58	1564 ± 127
13	2	2	−1	447 ± 49	423 ± 13	870 ± 62
14	2	2	0	346 ± 67	523 ± 23	869 ± 32
15	2	2	1	729 ± 40	291 ± 23	1020 ± 61
16	2	3	−1	32 ± 9	25 ± 0.6	57 ± 9
17	2	3	0	62 ± 3	98 ± 14	160 ± 14
18	2	3	1	94 ± 5	60 ± 2	154 ± 4
19	3	1	−1	735 ± 52	887 ± 151	1622 ± 157
20	3	1	0	671 ± 13	915 ± 42	1586 ± 57
21	3	1	1	617 ± 3	898 ± 29	1515 ± 32
22	3	2	−1	465 ± 47	496 ± 118	961± 128
23	3	2	0	454 ± 46	654 ± 59	1108 ± 102
24	3	2	1	543 ± 16	732 ± 6	1275 ± 22
25	3	3	−1	24 ± 15	24 ± 7	48 ± 22
26	3	3	0	16 ± 3	63 ± 11	79 ± 13
27	3	3	1	15 ± 6	77 ± 11	92 ± 14

SD: standard deviation (n = 3). * (A) extraction type, (B) solvent type, (C) sample/solvent ratio. TGAs refers to the combined amounts of α-solanine and α-chaconine.

**Table 3:** Comparison of the predicted values with the experimental results at statistically optimal extraction conditions.

Dependent Variable	Prediction	Lower Limit 95%	Upper Limit 95%	Experimental Value
α-solanine *	774	557	990	735
α-chaconine *	784	687	881	829
TGAs *	1559	1330	1788	1565

* Results are shown in mg/kg DW. TGAs refers to the combined amounts of α-solanine and α-chaconine.

**Table 4:** Validation parameters of the UAE-HPLC-DAD method for the quantification of α-solanine and α-chaconine in potato peel.

	Linearity					Method Accuracy ^d^		Method Precision ^d^		
	Linear Range (mg/L)	Calibration Line (R^2^) ^a^	LOD (mg/L) ^b^	LOQ (mg/L) ^c^		Recovery (% ± SD)	Mean Recovery (% ± SD)		Intra-Day (%RSD)	Inter-Day (%RSD)
α-solanine	1–100	y = 50.765x − 98.094 (0.9906)	0.30	1	Low	104 ± 5	103 ± 5	Low	5	4
					High	101 ± 6		High	6	13
α-chaconine	1–100	y = 51.854x − 15.205 (0.9910)	0.30	1	Low	100 ± 4	100 ± 4	Low	4	5
					High	100 ± 4		High	4	5

^a^ x = mg/L; ^b^ LOD: limit of detection estimated as 3 times the signal/noise ratio; ^c^ LOQ: limit of quantification estimated as 10 times the signal/noise ratio; ^d^ accuracy and precision were obtained by spiking samples at two concentration levels: low (200 mg/kg of α-solanine and α-chaconine) and high (400 of mg/kg α-solanine and α-chaconine).

**Table 5:** Moisture content and content of α-solanine, α-chaconine, and total glycoalkaloids (TGAs) in the peels of different potato varieties. TGAs refers to the combined amounts of α-solanine and α-chaconine.

Variety	α-Solanine (mg/kg DW)	α-Solanine (%)	α-Chaconine (mg/kg DW)	α-Chaconine (%)	TGAs (mg/kg DW)
Agata	307 ± 21	26	894 ± 35	74	1201 ± 51
Agria	234 ± 32	48	251 ± 41	52	485 ± 72
Amandine	1081 ± 27	39	1742 ± 8	61	2823 ± 33
Amaris	239 ± 26	42	327 ± 38	58	566 ± 20
Caesar	594 ± 23	43	803 ± 67	57	1397 ± 75
Colomba	217 ± 5	31	471 ± 41	69	681 ± 40
Evolution	262 ± 20	34	502 ± 52	66	764 ± 40
Frisia	247 ± 11	24	797 ± 70	76	1044 ± 68
Memphis	198 ± 6	23	680 ± 27	77	878 ± 28
Monalisa	306 ± 19	25	892 ± 29	75	1198 ± 40
Lady Amarilla	235 ± 12	37	393 ± 25	63	629 ± 31
Rudolph	1266 ± 78	44	1629 ± 44	56	2895 ± 89
Soprano	343 ± 12	39	546 ± 17	61	889 ± 28
Universa	193 ± 19	38	316 ± 16	62	509 ± 24
Vivaldi	143 ± 9	55	117 ± 9	45	260 ± 8

## Data Availability

The original contributions presented in the study are included in the article/supplementary material, further inquiries can be directed to the corresponding authors.

## Supplementary Materials

The following supporting information can be downloaded at: https://www.mdpi.com/article/10.3390/foods13050651/s1, Table S1: Potato peel varieties analyzed in this work and their specifications; *data obtained from the product labels.; Table S2: Values obtained from ANOVA analysis.
