# Supplementary material for: Development of a Green, Quick, and Efficient Method Based on Ultrasound-Assisted Extraction Followed by HPLC-DAD for the Analysis of Bioactive Glycoalkaloids in Potato Peel Waste
Source: Foods. 2024 Feb 21;13(5):651. doi: 10.3390/foods13050651 (PMC10930394; doi:10.3390/foods13050651)
Supplement: Supplementary file 1 [file foods-13-00651-s001.zip › foods-2877497-supplementary.pdf]

foods

Supplementary Materials

Development of a Green, Quick, and Efficient Method Based on Ultrasound-Assisted Extraction Followed by HPLC-DAD for the Analysis of Bioactive Glycoalkaloids in Potato Peel Waste

Isabel Martínez-García [1], Carlos Gaona-Scheytt [1], Sonia Morante-Zarcero [1,*] and Isabel Sierra [1,2,*]

[1] Departamento de Tecnología Química y Ambiental, E.S.C.E.T, Universidad Rey Juan Carlos, C/Tulipán s/n, 28933 Móstoles, Spain; isabel.martinezg@urjc.es (I.M.-G.); cf.gaona.2019@alumnos.urjc.es (C.G.-S.)

[2] Instituto de Tecnologías para la Sostenibilidad, Universidad Rey Juan Carlos, C/ Tulipán s/n, 28933 Móstoles, Spain

* Correspondence: sonia.morante@urjc.es (S.M.-Z.); isabel.sierra@urjc.es (I.S.)

Table S1. Potato peel varieties analyzed in this work and their specifications; *data obtained from the product labels.

Variety*	Origin*	Category*	Caliber (mm)*	Peel colour*	Use*	Moisture determined by freeze-drying (%)
**Agata**	France	I	50/80	Yellow	Boiling	83.15
**Agria**	Spain	I	50/75	Yellow	Frying	78.07
**Amandine**	France	I	40/65	Yellow	All elaborations	82.03
**Amarin**	Spain	I	50/80	Yellow	All elaborations	82.46
**Caesar**	France	I	50/80	Yellow	Friying, puree	84.33
**Colomba**	Spain	I	28/45	Yellow	Special for garnish	81.80
**Evolution**	Portugal	I	50/80	Red	All elaborations	82.86
**Frisia**	Spain	I	45/80	Yellow	All elaborations	83.66
**Memphis**	Spain	I	50/80	Red	All elaborations	85.40
**Monalisa**	France	I	50/80	Yellow	Frying, boiling	80.55
**Lady Amarilla**	Spain	I	50/80	Yellow	Frying	80.13
**Rudolph**	France	I	50/80	Red	All elaborations	82.97
**Soprano**	Spain	I	30/45	Yellow	Boiling, microwave	84.49
**Universa**	Spain	I	45/+	Yellow	Frying, oven, boiling, barbecue	79.95
**Vivaldi**	Spain	I	45/80	Yellow	All elaborations	82.49

Table S2. Values obtained from ANOVA analysis.

	Mean square	F-value	p-value
**TGAs**			
**A**	63168.4	3.597	0.0597
**B**	4.06734E6	231.631	0.0000*
**C**	152168	8.666	0.0123*
**AB**	96240.2	5.481	0.0096*
**AC**	66212.7	3.771	0.0536
**BC**	105768	6.023	0.0154*
**CC**	661.5	0.038	0.8494
**R^2 = 0.9775; R^2_{adj} = 0.9512; R^2_{pred} = 0.8717**			
**α-solanine**			
**A**	19856.3	1.270	0.3162
**B**	827242	52.883	0.0000*
**C**	51627.6	3.300	0.0943
**AB**	26981.4	1.725	0.2090
**AC**	68203.7	4.360	0.0377*
**BC**	37206.7	2.379	0.1349
**CC**	36296.3	2.320	0.1563
**R^2 = 0.9180; R^2_{adj} = 0.8223; R^2_{pred} = 0.5643**			
**α-chaconine**			
**A**	47084.1	14.905	0.0006*
**B**	1.21788E6	385.531	0.0000*
**C**	26526.7	8.397	0.0134*
**AB**	26760.4	8.471	0.0017*
**AC**	670.056	0.212	0.8118
**BC**	17523.4	5.547	0.0197*
**CC**	23688.2	7.499	0.0180*
**R^2 = 0.9863; R^2_{adj} = 0.9703; R^2_{pred} = 0.9165**			

*Significant at $p \leq 0.05$
